# Implementation of telemedicine consultations for people with mental health conditions in the community: a protocol for a systematic review

**DOI:** 10.12688/hrbopenres.13435.3

**Published:** 2022-10-19

**Authors:** Emer Galvin, Shane Desselle, Blánaid Gavin, Etain Quigley, Mark Flear, Ken Kilbride, Fiona McNicholas, Shane Cullinan, John Hayden

**Affiliations:** 1 School of Pharmacy and Biomolecular Sciences, Royal College of Surgeons in Ireland, Dublin, Ireland; 2 Touro University California, California, USA; 3 School of Medicine, University College Dublin, Dublin, Ireland; 4 National University of Ireland, Maynooth, Maynooth, Ireland; 5 Queen's University Belfast, Belfast, UK; 6 ADHD Ireland, Dublin, Ireland

**Keywords:** telemedicine, remote consultations, telepsychiatry, mental health, COVID-19

## Abstract

**Background:** The COVID-19 pandemic response has led to an exponential increase in the use and spread of telemedicine internationally. In community mental health care settings, telemedicine services were implemented within a few weeks, with little time for rigorous planning. Despite the reported acceptability of telemedicine by patients and clinicians, barriers to its implementation have come to light. There is now a need to investigate these barriers, and facilitators, as telemedicine begins to show potential promise beyond the pandemic. We propose a review that aims to identify the factors affecting the implementation of telemedicine consultations for patients with mental health conditions in the community.

**Methods:** A systematic review will be conducted and reported according to the PRISMA guidelines. Five electronic databases will be searched using a pre-defined search strategy from 2016 to 2021. Only studies of synchronous, interactive telemedicine consultations conducted via video, phone or live messaging between patients and providers will be included. Quantitative, qualitative and mixed methods studies will be eligible for inclusion. Only studies published in the English language will be included. Titles and abstracts will be screened by two reviewers. Full text articles will be screened by two reviewers. The methodological quality of studies will be assessed using the Mixed Method Appraisal Tool (MMAT) by two reviewers. Data will be extracted and tabulated to address the aims of the review. A narrative synthesis will be conducted and reported factors will be mapped to the domains of the Consolidated Framework for Implementation Research (CFIR).

**Conclusion:** By identifying the factors that influence the implementation of telemedicine consultations for patients with mental conditions in the community, consideration can be given to both barriers and facilitators that could be addressed in future mental health services planning.

**PROSPERO registration:** CRD42021273422 (04/10/2021)

## Introduction

The COVID-19 pandemic response caused swift, unprecedented changes to the delivery of healthcare. One such change was the rapid and widespread expansion of telemedicine services to comply with social distancing policies and reduce the spread of the virus ^1, 2^. Telemedicine is defined as the provision of healthcare at a distance through telecommunications and information technologies ^3^. Worldwide, longstanding regulatory barriers to telemedicine delivery were amended to facilitate its use during the pandemic ^4^. In community settings, telemedicine allowed for the continued and essential access to mental health services during the pandemic. This use of telemedicine in mental health care is often referred to as telemental health, and is defined as the use of telecommunication for the provision or support of mental health services over a distance ^5^. Heterogeneous definitions of telemedicine exist in the literature, so for the purposes of this review, telemedicine refers to live, synchronous remote consultations between provider and patient, using video, audio or live messaging modalities. These telemedicine consultations were chosen as the focus of this review as these types of consultations became commonplace during the pandemic ^6^, acting as a temporary replacement for in-person consultations.

In the past two decades, telemedicine in mental health care has emerged as a safe and acceptable method of improving mental health care access for those who are disenfranchised or hard-to-reach. Systematic reviews and meta-analyses have shown that treatment effects of telemental health are comparable to face-to-face mental health care ^7– 10^. There is also a body of evidence supporting the equivalence of telemental health to face-to-face care with regards to patient satisfaction and therapeutic alliance ^11– 13^. Despite a steady increase in use and evidence for its effectiveness, telemedicine constitutes a small portion of all mental health services prior to the pandemic ^14, 15^. Previous reviews of implementation factors have attributed the under-utilisation of telemedicine in mental health services to a number of reasons including strict licensure regulations and insurance policies that limit the reimbursement of telemedicine services ^16, 17^ and reluctance by clinicians ^18, 19^.

The rapid and highly variable adoption of telemedicine in mental health care settings during the COVID-19 pandemic has shed some light on this research- implementation gap. A number of challenges and barriers have come to light, including lack of technological infrastructure, privacy concerns, difficulty in establishing rapport and problems with conducting high quality assessments ^20, 21^. Despite these challenges, patients and clinicians have reported satisfaction with, and acceptance of, telemental health services during the pandemic ^22, 23^. Moreover, some patients and service users have expressed a desire to continue to use telemental health services in the future ^15, 24^; a view mirrored by policy makers and mental health professionals ^25, 26^. To harness the possible potential of telemedicine in future mental health care, a systematic exploration of the factors that affect successful telemedicine implementation in community mental health services is now needed. Identifying these factors, both enabling and hindering, will help ensure its acceptable and effective use going forward.

To gain a thorough understanding of the factors that influence the implementation of telemedicine into community mental health services, a strong theoretical foundation to guide interpretation of these factors is required. Various theories, models and frameworks have been developed in the area of implementation research to understand the determinants of translating research into practice ^27^. One such framework is the Consolidated Framework for Implementation Research (CFIR) ^28^. The CFIR consolidates the various terms used in implementation research into five domains considered to be important moderators or mediators of implementing practice into research ^28^. These five domains include 1) intervention characteristics, 2) outer setting, 3) inner setting, 4) characteristics of individuals and 5) process. This framework has been used to assess the implementation of evidence-based practices in health and mental health settings ^29, 30^. The CFIR encompasses terms and concepts from numerous implementation frameworks and has therefore been selected to map the findings of this review. Its comprehensiveness allows for the categorization of various implementation factors across a variety of study designs and patient populations ^28^, which is relevant to this review.

To our knowledge, no systematic review exists of the factors that affect implementation of telemedicine for patients with mental health conditions in community settings. In consideration of the swift and variable adoption of telemedicine in the COVID-19 pandemic, a number of overlooked factors may emerge as likely determinants of successful telemedicine implementation. This review is pertinent as mental health professionals and policy makers are now interested in the continued, long-term use of telemedicine in mental services beyond the COVID-19 pandemic ^14, 31^. Moreover, this review is particularly relevant given the unprecedented rise in mental health problems and increased demand on mental health services arising from the pandemic ^32, 33^. It is crucial that we now take stock of the available evidence regarding the challenges and successes of implementing telemedicine to identify key factors for its acceptable adoption into routine community mental health care. Subsequently, consideration can be given to solutions that address these factors by stakeholders involved in mental health services planning.

The primary aim of this review is to identify, summarise and interpret the key factors affecting the implementation of telemedicine consultations for patients with mental health conditions in the community. The secondary aim is to map these factors to the domains of the CFIR.

## Protocol

This protocol has been prepared following the PRISMA-P (Preferred Reporting Items for Systematic Reviews and Meta-Analyses for Protocols) 2015 checklist ^34, 35^ (See *Reporting Guidelines*). The protocol is registered on the International Prospective Register of Systematic Reviews (PROSPERO) as CRD42021273422. A systematic review design was chosen to synthesise the available literature. As the scope of the research question is narrow in nature and we are interested in synthesising empirical research, a systematic review approach was deemed appropriate ^36^.

### Eligibility criteria

Studies will be selected for inclusion in the systematic review according to the following PEO acronym criteria (population, exposure and outcome).

**Population.** The population will include adults and children (aged < 18 years) with a diagnosis of a mental disorder or in receipt of care from a mental health professional (e.g. psychiatrist, psychotherapist, counsellor). Mental disorders included will be in accordance with the International Classification of Diseases (ICD-11) criteria for mental and behavioural disorders (WHO, 2019). We will include common mental disorders such as depression, generalised anxiety disorder, social anxiety disorder, obsessive-compulsive disorder and post-traumatic stress disorder. Severe mental disorders such as schizophrenia and other psychotic disorders, and bipolar disorder will also be included. Neurocognitive disorders, such as dementia, perinatal mental disorders, disorders associated with substance abuse, disorders associated with stress, eating disorders and neurodevelopmental disorders, such as ADHD, will be included. Studies that focus on people learning difficulties, intellectual disabilities and people with mental health problems secondary due to physical illness will also be included. We will include studies which include health care professionals (e.g. doctors, nurses, allied health professionals) involved in the provision of mental health care via telemedicine to patients with the above conditions or without a formal psychiatric diagnosis.

**Exposure.** Studies evaluating synchronous, live, interactive telemedicine consultations between patient and provider, including video-conferencing, telephone and live-messaging only will be included. Studies will be restricted to those that use synchronous (real-time) consultations between a patient and one, or more, health care professionals. Studies will be excluded if they use asynchronous methods, in which the healthcare professional and patient do not interact in real-time, such as email communications. Studies exploring telemedicine consultations between practitioners, such as when a health care professional seeks advice from another practitioner, will be excluded.

**Outcome.** Studies with data on the factors that affect the implementation of telemedicine consultations for people with mental health conditions in the community will be included. Studies that explore the views and experiences of patients, parents/carers of patients and/or healthcare professionals on the implementation of telemedicine consultations for patients with mental health conditions in the community are eligible for inclusion. Studies only exploring anticipated or hypothetical views will be excluded. 

**Study design.** Qualitative, quantitative and mixed-methods studies will be included in the review. Systematic reviews, meta-analyses and study protocols will be excluded as they do not contain primary research. We will only include full-text studies reported in the English language, due to constraints on resources to translate studies. Grey literature will be excluded, including non-peer reviewed articles, conference proceedings, case reports, editorials, opinion papers and letters. We have decided to exclude grey literature because of the variability in quality, peer review, supplication in full-text reviews and the need for prompt publication of findings.

**Setting.** Only studies in primary care, community or outpatient settings will be included. Studies in inpatient hospital settings, residential care homes, and prisons will be excluded. 

### Information sources and search strategy

An initial search of the MEDLINE database will be conducted to identify articles on the topic. Five electronic databases will be systematically searched for studies published between 2016 and 2021; MEDLINE (PubMed), Embase, Web of Science, CINAHL and APA PsycINFO. This timeframe (2016 to 2021) was chosen to include studies prior to, and during, the pandemic to ensure a comprehensive review of implementation-related factors. We chose not to include studies before this time point as the use of technology has grown dramatically in the past five years and the context in which telemedicine was implemented previously may be largely different to what is relevant in today’s context. The search strategy will contain terms pertaining to mental health conditions, telemedicine, community settings and implementation. See *Extended Data* for the PubMed sample search strategy ^35^. Forward and backward citation searches of included articles will also be conducted.

### Study selection

Two reviewers (EG and JH) will independently screen titles and abstracts of all articles in order to identify studies that meet the inclusion and exclusion criteria. The full texts of all selected articles will be collected and examined by two reviewers, independent of each other (EG and JH). Any disagreements will be mediated through a third reviewer (SC). Duplicates will be excluded. A PRISMA flow chart will display the articles examined at each stage, detailing the number of papers included and excluded and reasons for exclusions.

### Data extraction and management

The following data items will be extracted from all studies by one reviewer (EG):

1. Author(s)
2. Publication year
3. Date of data collection
4. Country of publication
5. Study aims
6. Population characteristics
7. Sample size
8. Study design
9. Telemedicine consultation type (video/phone/messaging)
10. Key relevant outcomes relating to the research question

Due to restraints on resources that prevent independent extraction of data by two reviewers, one reviewer (EG) will extract the data while a second reviewer (JH) will review a random 20% of the extracted data for accuracy. Any disagreements between the reviewers relating to the extracted data will aimed to be resolved through consensus. If the two reviewers are unable to come to a consensus, a third reviewer (SC) will be consulted. If the third reviewer is unable to arbitrate, the study authors will be contacted to seek clarification on the issue. If this step is unsuccessful, the disagreement will be recorded and reported in the review ^37^. In addition, reported factors will be extracted and categorised into the five domains of the CFIR by one reviewer (EG). Microsoft Excel software will be used to organize the extracted data. Any uncertainties regarding data will be resolved by attempting to contact study authors via email. Selected articles will be stored and managed using EndNote X9 Reference Manager Library.

### Quality assessment

Two reviewers (EG and JH) will independently assess the methodological quality of the included studies using the Mixed-Methods Appraisal Tool (MMAT) (Hong *et al.*, 2018). Any discrepancies between study assessments will be discussed and resolved. Studies will not be excluded based on quality.

### Data synthesis

One reviewer (EG) will perform a thematic synthesis on the extracted qualitative findings following Thomas and Harden’s ^38^ guidance. This will involve coding the qualitative findings and analysing the identifying themes relating to the implementation factors. This inductive approach was chosen to ensure all relevant implementation factors were identified, including those that may not fit in an existing framework. This synthesis will be verified by a second reviewer (JH). Quantitative findings will be summarised narratively. One reviewer (EG) will map the reported factors to the five domains of the CFIR (outer-setting, inner-setting, intervention, individual or process) and present them in a table format. We will use the GRADE Confidence in the Evidence from Reviews of Qualitative Research (CERQual) to rate the overall confidence in the qualitative evidence synthesis and the narrative summaries of the quantitative data ^39^. Any notable similarities and differences in implementation factors between studies conducted before, and during, the pandemic will be discussed narratively.

## Study status

The database searches were completed in August 2021. Full-text screening was completed in November 2021. It is anticipated that this review will be completed in March 2022.

## Discussion

The rapid and unprecedented uptake of telemedicine since the COVID-19 pandemic has brought this modality of healthcare to the forefront of health services research. The potential of telemedicine to increase access to mental health services and alleviate the mental health burden is promising. However, challenges to its implementation in current mental health services are still present, such as the potential exacerbation of inequalities and technological barriers ^40^. This review will aim to shed light on the factors that may enable or hinder the implementation of telemedicine for people with mental health conditions in the community. By identifying and interpreting these factors, consideration can be given to solutions that can optimise remotely-delivered mental health care for patients in the community.

A strength of this review is the use of a determinant framework, the CFIR, to map the findings to as it aids the transferability and generalisability of findings to other implementation studies ^28^. Furthermore, the CFIR incorporates concepts from multiple implementation theories which makes it less likely that important factors will be overlooked ^28^. A number of steps will be undertaken to minimise the risk of meta-biases in the review. Firstly, the systematic review will be conducted and reported using the PRISMA guidelines ^41^. Secondly, the risk of selection bias will be minimised by two independent reviewers performing title and abstract, and full-text screening. Thirdly, two independent reviewers will appraise the quality of the included studies. A limitation of the review is that the studies will be restricted to those conducted in the English language due to financial constraints which may bias the results. In addition, excluding unpublished studies and grey literature may increase the risk of publication bias. Another potential limitation is the heterogeneity of implementation factors and patient populations reported in the included studies. This may make it difficult to synthesise and compare the findings from each study. A final limitation is the use of one reviewer to extract data which may increase the risk of errors at this stage.

Despite these limitations, this review will be an important and timely contribution to understanding how to improve the implementation of telemedicine for patients with mental health conditions in the community. Findings of the review may advise policy makers and other stakeholders involved in the implementation of telemedicine services, informing their future development. The results of the systematic review will be reported in a peer-reviewed journal, presented at national and international conferences and included in a PhD thesis.

## Data Availability

No underlying data are associated with this article. Open Science Framework: Implementation of telemedicine consultations for people with mental health conditions in the community: a protocol for a systematic review. https:///doi.org/10.17605/OSF.IO/CKBEQ ^35^. This project contains the following extended data: Sample Search Strategy PubMed.pdf (PubMed Search Strategy) Open Science Framework: PRISMA-P checklist for "Implementation of telemedicine consultations for people with mental health conditions in the community: a protocol for a systematic review", https:///doi.org/10.17605/OSF.IO/CKBEQ ^35^. Data are available under the terms of the Creative Commons Zero "No rights reserved" data waiver (CC0 1.0 Public domain dedication).
