# Supplementary material for: Implementation of telemedicine consultations for people with mental health conditions in the community: a protocol for a systematic review
Source: HRB Open Res. 2022 Oct 19;4:125. Originally published 2021 Dec 6. [Version 3] doi: 10.12688/hrbopenres.13435.3 (PMC9627101; doi:10.12688/hrbopenres.13435.3)
Supplement: Peer review history

# Peer review: Reviewer response for version 3

**DOI:** 10.21956/hrbopenres.14907.r33079

**Published:** 2022-11-01

**Authors:** Shalini Lal, Rossana Peredo

**Affiliations:** 1 School of Rehabilitation, University of Montreal, Montreal, QC, Canada; 2 University of Montreal , Montreal, Canada

Thank you for your responses. We consider that the changes are satisfactory – the authors have provided details about the second reviewer's role, and they also acknowledged the limitation of having only one reviewer for data extraction.

Is the study design appropriate for the research question?

Yes

Is the rationale for, and objectives of, the study clearly described?

Partly

Are sufficient details of the methods provided to allow replication by others?

Partly

Are the datasets clearly presented in a useable and accessible format?

Not applicable

Reviewer Expertise:

NA

We confirm that we have read this submission and believe that we have an appropriate level of expertise to confirm that it is of an acceptable scientific standard.

# Peer review: Reviewer response for version 3

**DOI:** 10.21956/hrbopenres.14907.r33078

**Published:** 2022-10-21

**Authors:** Karen Jean Day

**Affiliations:** 1 School of Population Health, The University of Auckland, Auckland, New Zealand

Thank you for making the requested adjustments.

Is the study design appropriate for the research question?

Yes

Is the rationale for, and objectives of, the study clearly described?

Yes

Are sufficient details of the methods provided to allow replication by others?

Yes

Are the datasets clearly presented in a useable and accessible format?

Yes

Reviewer Expertise:

Telehealth, mental health and telemedicine, e-mental health, digital health, health informatics.

I confirm that I have read this submission and believe that I have an appropriate level of expertise to confirm that it is of an acceptable scientific standard.

# Peer review: Reviewer response for version 2

**DOI:** 10.21956/hrbopenres.14827.r32280

**Published:** 2022-06-23

**Authors:** Karen Jean Day

**Affiliations:** 1 School of Population Health, The University of Auckland, Auckland, New Zealand

Thank you for making changes to your protocol in response to my review.

Is the study design appropriate for the research question?

Yes

Is the rationale for, and objectives of, the study clearly described?

Yes

Are sufficient details of the methods provided to allow replication by others?

Yes

Are the datasets clearly presented in a useable and accessible format?

Yes

Reviewer Expertise:

Telehealth, mental health and telemedicine, e-mental health, digital health, health informatics.

I confirm that I have read this submission and believe that I have an appropriate level of expertise to confirm that it is of an acceptable scientific standard.

# Peer review: Reviewer response for version 2

**DOI:** 10.21956/hrbopenres.14827.r32281

**Published:** 2022-06-21

**Authors:** Shalini Lal, Rossana Peredo

**Affiliations:** 1 School of Rehabilitation, University of Montreal, Montreal, QC, Canada; 2 University of Montreal , Montreal, Canada

Thank you to the authors for their detailed answers. The authors have addressed the concerns we raised in our peer review report appropriately, with the exception of one comment regarding methodology: 

 " **Comment 5:** *Methods: Authors should consider piloting the data extraction process with two reviewers, and include a second reviewer to validate the data extraction to ensure consistency and comprehensiveness in the data extraction process, and to minimize biases and human error.*

**Response:** Thank you for this comment. We agree that independent data extraction from two reviewers would help to improve consistency and comprehensiveness. Due to resource and time constraints, a second reviewer will now extract data items from a random 20% sample of studies to minimize bias. The *Data extraction and management *section of the manuscript has been amended to reflect this change."

 The response clarifies the reason for using only one reviewer for data extraction, however, we consider that this should be acknowledged in the discussion section as part of the limitations. Also, it is unclear if the second reviewer will extract data from a random 20% sample (pg. 5, line 22) as part of a pilot or a validation process. We suggest clarifying and describing the details of this process. Will the second reviewer extract the data before or after the first reviewer completes the entire data extraction? How will the two extractions be compared? What will happen in case of disagreement? 

 Finally, a minor editorial comment: there seems to be a typo: conduct vs contact, in the following phrase: “Any uncertainties regarding data will be resolved by attempting to conduct study authors via email” (pg. 5)”.

Is the study design appropriate for the research question?

Yes

Is the rationale for, and objectives of, the study clearly described?

Partly

Are sufficient details of the methods provided to allow replication by others?

Partly

Are the datasets clearly presented in a useable and accessible format?

Not applicable

Reviewer Expertise:

NA

We confirm that we have read this submission and believe that we have an appropriate level of expertise to confirm that it is of an acceptable scientific standard, however we have significant reservations, as outlined above.

# Response

**Published:** 2022-09-23

**Authors:** Emer Galvin

**Affiliations:** Royal College of Surgeons in Ireland, Ireland

**Comment:**

 "Comment 5: Methods: Authors should consider piloting the data extraction process with two reviewers, and include a second reviewer to validate the data extraction to ensure consistency and comprehensiveness in the data extraction process, and to minimize biases and human error.

 Response: Thank you for this comment. We agree that independent data extraction from two reviewers would help to improve consistency and comprehensiveness. Due to resource and time constraints, a second reviewer will now extract data items from a random 20% sample of studies to minimize bias. The Data extraction and management section of the manuscript has been amended to reflect this change."

 The response clarifies the reason for using only one reviewer for data extraction, however, we consider that this should be acknowledged in the discussion section as part of the limitations.

**Response:**

 Sincere thanks for your comments. We have acknowledged the limitation of using a single reviewer for data extraction in the *Discussion* section:

**A final limitation is the use of one reviewer to extract data which may increase the risk of errors at this stage.**

**Comment:**

 Also, it is unclear if the second reviewer will extract data from a random 20% sample (pg. 5, line 22) as part of a pilot or a validation process. We suggest clarifying and describing the details of this process. Will the second reviewer extract the data before or after the first reviewer completes the entire data extraction? How will the two extractions be compared? What will happen in case of disagreement?

**Response:**

 Thank you for this comment. We have sought guidance on the data extraction process from Taylor, Mahtani, and Aronson (2021) and have clarified the details of data extraction in the manuscript. Please see changes to the *Data extraction and management section* below:

**Due to restraints on resources that prevent independent extraction of data by two reviewers, one reviewer (EG) will extract the data while a second reviewer (JH) will review a random 20% of the extracted data for accuracy. Any disagreements between the reviewers relating to the extracted data will aimed to be resolved through consensus. If the two reviewers are unable to come to a consensus, a third reviewer (SC) will be consulted. If the third reviewer is unable to arbitrate, the study authors will be contacted to seek clarification on the issue. If this step is unsuccessful, the disagreement will be recorded and reported in the review** **(Taylor, Mahtani & Aronson, 2020). **In addition, reported factors will be extracted and categorised into the five domains of the CFIR by one reviewer (EG).

 Reference: Taylor KS, Mahtani KR, Aronson JK. Summarising good practice guidelines for data extraction for systematic reviews and meta-analysis. *BMJ Evidence-Based Medicine 2021*;26:88-90.

**Comment:**

 Finally, a minor editorial comment: there seems to be a typo: conduct vs contact, in the following phrase: “Any uncertainties regarding data will be resolved by attempting to conduct study authors via email” (pg. 5)”.

**Response:**

 Thank you for spotting this error. We have carefully reviewed the manuscript for typographical errors.

# Peer review: Reviewer response for version 1

**DOI:** 10.21956/hrbopenres.14639.r30996

**Published:** 2022-03-07

**Authors:** Shalini Lal, Rossana Peredo

**Affiliations:** 1 School of Rehabilitation, University of Montreal, Montreal, QC, Canada; 2 University of Montreal , Montreal, Canada

The authors present a protocol of a systematic review that aims to identify and summarize the key factors that affect the implementation of telemedicine based on recent literature. This purpose is particularly timely given that the use of telemedicine has increased exponentially especially during the pandemic, along with the number of publications on this subject. In general, the protocol is aligned with guidelines for systematic review protocols, and the consideration of theory to guide the interpretation of the factors is a strength of the review. The following points would be important to consider to strengthen the protocol:

1. Introduction: It would be good to provide some indication of existing reviews on the topic to help strengthen and better situate this review in relation to what is already known on the topic of implementation factors pertaining to telemedicine – especially given decades of research existing on the topic. There are a number of reviews related to the current review that could be considered, some that are focused on implementation factors pertaining to specific mental health conditions (e.g., Kruse *et al.* 2018 ^1^), others that are focused on implementation factors in telemedicine (that include as part of the included studies those focused on mental health field), and others that review telemedicine in mental health services more broadly that include a component addressing implementation factors.
2. Introduction: Clarify the rationale for including phone-based telemedicine, video, and messaging, as the technologies are different, with implementation factors likely to differ at least in part. Moreover, phone-based telemedicine has a longer, multi-decade research history including implementation research, and thus the rationale for the need to include this format of telemedicine in this current review is unclear.
3. Methods: Given the focus on Covid-19 in the abstract and introduction, it would be good to elaborate the justification for choosing 2016 (vs. 2019, vs. 2015 etc.) as publication date restriction. Moreover, the objective of a systematic review is to cover all available literature, as such, any restrictions should be well justified.
4. Methods: Involving two reviewers during screening and selection process, and piloting the process to ensure consistency taken in the study selection process is generally considered important to minimize bias and human error. Please clarify why a second reviewer will only be involved in 20% of random sample validation and this only at the stage of full text article retrieval.
5. Methods: Authors should consider piloting the data extraction process with two reviewers, and include a second reviewer to validate the data extraction to ensure consistency and comprehensiveness in the data extraction process, and to minimize biases and human error.
6. Method: It would be good to clarify how authors will handle the studies pre-pandemic versus during pandemic, as the implementation factors may overlap but also differ.
7. Discussion: It would be good to reduce the repetition in the first paragraph of the discussion with what is written in the introduction section of the paper

Is the study design appropriate for the research question?

Yes

Is the rationale for, and objectives of, the study clearly described?

Partly

Are sufficient details of the methods provided to allow replication by others?

Partly

Are the datasets clearly presented in a useable and accessible format?

Not applicable

Reviewer Expertise:

NA

We confirm that we have read this submission and believe that we have an appropriate level of expertise to confirm that it is of an acceptable scientific standard, however we have significant reservations, as outlined above.

## References

[1] : Factors influencing the adoption of telemedicine for treatment of military veterans with post-traumatic stress disorder. *J Rehabil Med* .2018;50(5) : 10.2340/16501977-2302 385-392 10.2340/16501977-2302 29700551

# Response

**Published:** 2022-05-31

**Authors:** Emer Galvin

**Affiliations:** Royal College of Surgeons in Ireland, Ireland

Dear Dr Shalini Lal & Dr Rossana Peredo,

 Thank you very much for your comments. We appreciate your valuable comments and have responded to each one below.

**Comment:** *Introduction: It would be good to provide some indication of existing reviews on the topic to help strengthen and better situate this review in relation to what is already known on the topic of implementation factors pertaining to telemedicine – especially given decades of research existing on the topic. There are a number of reviews related to the current review that could be considered, some that are focused on implementation factors pertaining to specific mental health conditions (e.g., Kruse et al. 20181), others that are focused on implementation factors in telemedicine (that include as part of the included studies those focused on mental health field), and others that review telemedicine in mental health services more broadly that include a component addressing implementation factors.*

**Response:** Thank you for this comment. We acknowledge that previous reviews in the broader area of telemental health have been conducted to examine implementation barriers and facilitators. Given the increase in telemedicine adoption during the pandemic, it is likely that new implementation factors may emerge, or indeed previous challenges and benefits may be emphasised. We have amended the *Introduction* section to highlight existing reviews, which can be seen in bold below:

**Previous reviews of implementation factors have attributed** the under-utilisation of telemedicine in mental health services to a number of reasons including strict licensure regulations and insurance policies that limit the reimbursement of telemedicine services ^15, 16^ and reluctance by clinicians ^17, 18^. (pg. 3)

**Comment:** *Introduction: Clarify the rationale for including phone-based telemedicine, video, and messaging, as the technologies are different, with implementation factors likely to differ at least in part. Moreover, phone-based telemedicine has a longer, multi-decade research history including implementation research, and thus the rationale for the need to include this format of telemedicine in this current review is unclear* **.**

**Response: **Thank you for this comment. We chose to focus on live, synchronous methods of telemedicine as these were most commonly used as a replacement for in-person consultations during the pandemic. We chose to focus on these three modalities of telemedicine consultations which often include both phone and video (and sometimes live-messaging) as health care professionals and patients use these interchangeably, and sometimes in combination with each other. In other cases, video consultations may be used without visual input which would have similarities to phone consultations. We have amended the manuscript to provide additional rationale for our decision to focus on these live, synchronous modalities of telemedicine. Please see below the change in bold:

**These telemedicine consultations were chosen as the focus of this review as these types of consultations became commonplace during the pandemic ^1^, acting as a temporary replacement for in-person consultations. **(pg. 3)

**Comment:** *Methods: Given the focus on Covid-19 in the abstract and introduction, it would be good to elaborate the justification for choosing 2016 (vs. 2019, vs. 2015 etc.) as publication date restriction. Moreover, the objective of a systematic review is to cover all available literature, as such, any restrictions should be well justified*

**Response:** Thank you for this comment. We chose the five-year time restriction for a number of reasons. Firstly, a scoping search of the literature revealed a low number of studies on remote mental health consultations before the five-year time point (i.e. consultations that mirror those that were conducted during the pandemic). Secondly, the use of technology has increased in recent years, which means that telemental health before this time point was conducted in a different context and implementation factors may not be relevant to the current technology and health landscape. We have amended the manuscript to include further rationale for this time restriction. Please see the amendment in bold below:

**We chose not to include studies before this time point has the use of technology has grown dramatically in the past five years and the context in which telemedicine was implemented previously may be largely different to what is relevant in today’s context. **(pg. 4)

**Comment:** *Methods: Involving two reviewers during screening and selection process, and piloting the process to ensure consistency taken in the study selection process is generally considered important to minimize bias and human error. Please clarify why a second reviewer will only be involved in 20% of random sample validation and this only at the stage of full text article retrieval.*

**Response:** Thank you for this comment. The second reviewer will now review 100% of full-text articles, independent to the first reviewer. The *Study selection* section of the manuscript has been amended to include this change. Please see the change in bold below:

 The full texts of all selected articles will be collected and examined **by two reviewers, independent of each other (EG and JH). **(pg. 4)

**Comment:** *Methods: Authors should consider piloting the data extraction process with two reviewers, and include a second reviewer to validate the data extraction to ensure consistency and comprehensiveness in the data extraction process, and to minimize biases and human error.*

**Response:** Thank you for this comment.  We agree that independent data extraction from two reviewers would help to improve consistency and comprehensiveness. Due to resource and time constraints, a second reviewer will now extract data items from a random 20% sample of studies to minimize bias. The *Data extraction and management *section of the manuscript has been amended to reflect this change.

**Comment:** *Method: It would be good to clarify how authors will handle the studies pre-pandemic versus during pandemic, as the implementation factors may overlap but also differ.*

**Response: **Thank you for this comment. We plan to synthesis all included studies in one analysis. However, we plan to highlight similarities and differences narratively in the *Results* and *Discussion* section of the review. We have amended the *Data synthesis *section of manuscript to clarify this. Please see the change in bold below:

**Any notable similarities and differences in implementation factors between studies conducted before, and during, the pandemic will be discussed narratively. **(pg. 5)

**Comment:** *Discussion: It would be good to reduce the repetition in the first paragraph of the discussion with what is written in the introduction section of the paper*

**Response:** Thanks for this comment. We have made some amendments in the *Discussion* section to reduce repetition from the Introduction section.

# Peer review: Reviewer response for version 1

**DOI:** 10.21956/hrbopenres.14639.r31312

**Published:** 2022-02-14

**Authors:** Karen Jean Day

**Affiliations:** 1 School of Population Health, The University of Auckland, Auckland, New Zealand

Thank you for the opportunity to review this literature review protocol about telemedicine consultations for people with mental health conditions in the community. Overall, it is very well-written, clearly stating the review purpose, what is planned, how it will be done, and what outcomes are expected. It is good to see the use of frameworks to strengthen the literature review. The protocol could be improved in the following ways.

 In the introduction, first paragraph, the authors indicate that the study is about telemedicine, and they provide an excellent definition of the concept. The link between mental health, psychiatry, and mental health professionals is not as clear. Is this review about general mental health and all those healthcare professionals who provide services, or is it focused on psychiatry? It appears that the review is about the former, but the boundaries are blurred in ways that may create confusion. Who are the mental health professionals who are not psychiatrists, e.g., do psychiatric nurses, psychologists, counselors, social workers qualify as ‘mental health professionals’? I ask this question because you later say (page 4, section on population), “ *We will include studies which include health care professionals (e.g. doctors, nurses, allied health professionals) involved in the provision of telemedicine to patients with the above conditions*.” This statement introduces ambiguity about who the healthcare professionals are who you assume to provide mental health care via telemedicine. It would be good if you could remove this ambiguity throughout the paper.

 The choice of a systematic review appears to be a good one. However, one cannot judge this without some discussion about alternative types of reviews that you may have considered and rejected for various reasons. The article by Pare *et al. *(2015) ^1^ can help you briefly write about alternative approaches that you have rejected to strengthen your decision for a systematic review.

 Your choice to exclude conference proceedings may not work in your favour. There are many useful digital health- and telehealth-related conference proceedings that are peer-reviewed and published and you may end up with an unhelpful bias. Many digital health practitioners attend these conferences and are influenced in their practice by what they learn from the conferences. Conversely, many of them don’t have access to journal publications that are behind a subscription paywall. IEEE is a highly regarded conference and their proceedings are often given the same value as journal publications. Telehealth and digital health conferences, such as MedInfo, are published in “Studies in Health Technology and Informatics”. It would be a shame to exclude these peer-reviewed papers that influence practice and policy. You plan to do a quality review of the included publications to protect the quality of your review, so you can still report on conference proceedings without compromising the review. If you choose to not take this advice, please indicate why in your final published protocol.

Is the study design appropriate for the research question?

Yes

Is the rationale for, and objectives of, the study clearly described?

Yes

Are sufficient details of the methods provided to allow replication by others?

Yes

Are the datasets clearly presented in a useable and accessible format?

Yes

Reviewer Expertise:

Telehealth, mental health and telemedicine, e-mental health, digital health, health informatics.

I confirm that I have read this submission and believe that I have an appropriate level of expertise to confirm that it is of an acceptable scientific standard, however I have significant reservations, as outlined above.

## References

[1] : Synthesizing information systems knowledge: A typology of literature reviews. *Information & Management* .2015;52(2) : 10.1016/j.im.2014.08.008 183-199 10.1016/j.im.2014.08.008

# Response

**Published:** 2022-05-31

**Authors:** Emer Galvin

**Affiliations:** Royal College of Surgeons in Ireland, Ireland

Dear Dr Karen Day,

 Sincere thanks for your comments. We appreciate your valuable input and have responded to your comments below. Please see the amendments in the revised paper.

**Comment:** *In the introduction, first paragraph, the authors indicate that the study is about telemedicine, and they provide an excellent definition of the concept. The link between mental health, psychiatry, and mental health professionals is not as clear. Is this review about general mental health and all those healthcare professionals who provide services, or is it focused on psychiatry? It appears that the review is about the former, but the boundaries are blurred in ways that may create confusion.*

**Response:** Thanks for this comment. This review is about general mental health and all those healthcare professionals who provide mental health services. We acknowledge that the various terms (i.e. telepsychiatry) may cause some confusion so we have changed any mention of the term “telepsychiatry” to “telemental health” in the manuscript in an attempt to overcome this ambiguity.

**Comment:** *Who are the mental health professionals who are not psychiatrists, e.g., do psychiatric nurses, psychologists, counselors, social workers qualify as ‘mental health professionals’? I ask this question because you later say (page 4, section on population), “We will include studies which include health care professionals (e.g. doctors, nurses, allied health professionals) involved in the provision of telemedicine to patients with the above conditions.” This statement introduces ambiguity about who the healthcare professionals are who you assume to provide mental health care via telemedicine. It would be good if you could remove this ambiguity throughout the paper.*

**Response:** Thank you for this comment. We will include studies of healthcare professionals involved in the provision of mental health services via telemedicine. We have amended the *Population *section of the manuscript to attempt to clarify any ambiguity regarding this inclusion criteria. We have also amended the *Population *section to include mental health professionals who provide mental health services to people without a formal diagnosis of a mental disorder. Please see amendments to the *Population *section in bold below:

 The population will include adults and children (aged < 18 years) with a diagnosis of a mental disorder or in receipt of care from **a mental health professional (e.g. psychiatrist, psychotherapist, counsellor). **(pg. 4)

 We will include studies which include healthcare professionals (e.g. doctors, nurses, psychologists, counsellors, psychotherapists) involved in the provision of **mental health care via **telemedicine to patients with the above conditions **or without a formal psychiatric diagnosis.** (pg. 4)

**Comment:** *The choice of a systematic review appears to be a good one. However, one cannot judge this without some discussion about alternative types of reviews that you may have considered and rejected for various reasons. The article by Pare et al. (2015)1 can help you briefly write about alternative approaches that you have rejected to strengthen your decision for a systematic review.*

**Response:** Thank you for this comment and for attaching that reference. We chose not to do a scoping review as the scope of the questions is quite narrow, which is more suited to a systematic review. Furthermore, a scoping review is relevant when attempting to understand the initial size and nature of the available literature. This type of methodology is not suited to this review where previous reviews have been conducted in the broader area of telemental health implementation, prior to the pandemic. We also intended to include only empirical research which is more suited to a systematic review design, than a scoping review design. We decided not to conduct a meta-analysis as we are interested in narratively presenting our findings. It is unlikely that a meta-analysis would be possible as the outcome of interest (e.g. implementation factors) does not have standardised outcome measure that would be comparable across studies. We have included additional sentences in the *Protocol *section of the manuscript outlining our decision. Please see changes in bold below:

**A systematic review design was chosen to synthesise the available literature. As the scope of the research question is narrow in nature and we are interested in synthesising empirical research, a systematic review approach was deemed appropriate ^2^. **(pg. 3)

**Comment:** *Your choice to exclude conference proceedings may not work in your favour. There are many useful digital health- and telehealth-related conference proceedings that are peer-reviewed and published and you may end up with an unhelpful bias. Many digital health practitioners attend these conferences and are influenced in their practice by what they learn from the conferences. Conversely, many of them don’t have access to journal publications that are behind a subscription paywall. IEEE is a highly regarded conference and their proceedings are often given the same value as journal publications. Telehealth and digital health conferences, such as MedInfo, are published in “Studies in Health Technology and Informatics”. It would be a shame to exclude these peer-reviewed papers that influence practice and policy. You plan to do a quality review of the included publications to protect the quality of your review, so you can still report on conference proceedings without compromising the review. If you choose to not take this advice, please indicate why in your final published protocol.*

**Response:** Thank you very much for your comment. We acknowledge that IEEE is a useful resource, and includes peer-reviews papers. We will use this resource to help shape recommendations in the *Discussion* section of the final review. We acknowledge that excluding conference proceedings may run the risk of excluding potentially relevant studies. We have decided to exclude this “grey literature” because of the variability in quality, peer review, supplication in full-text reviews, and the need for prompt publication of findings. We recognise the potential limitation of excluding these studies and this is reflected in the *Discussion* section. We will also acknowledge this as a limitation on the final review. We have amended the *Study design *section of the protocol to clarify why we have excluded grey literature. Please see the amendment in bold below.

**We have decided to exclude grey literature because of the variability in quality, peer review, supplication in full-text reviews, and the need for prompt publication of findings. **(pg. 4).
